# The Algorhytm: FRAX Brazil

**DOI:** 10.1055/s-0039-1695027

**Published:** 2019-08

**Authors:** Ben Hur Albergaria, Francisco José Albuquerque Paula

**Affiliations:** 1 Department of Social Medicine, Universidade Federal do Espírito Santo, Vitória, ES, Brazil; 2 Faculty of Medicine, Universidade de São Paulo, Ribeirão Preto, SP, Brazil

The World Health Organization (WHO) defines osteoporosis as a skeletal disorder characterized by low bone mass combined with microarchitetural deterioration of the bone, leading to bone fragility and increased susceptibility to fracture. Its clinical relevance relies on the emerging fracture rate, as well as on the morbidity and mortality associated with hip fractures. Moreover, the WHO objectively defines osteoporosis based on bone mineral density (BMD, [that is, when BMD is ≤ -2.5 standard deviations [SD] below peak bone mass [T-score], as assessed by dual X-ray absorptiometry [DXA]). A T-score between -1.0 and -2.5 means an intermediary condition of bone loss, which is called osteopenia. There is no doubt that the risk of fracture increases significantly with decreasing BMD. In spite of this, it is well known that osteoporotic fractures occur across a wide spectrum of BMD intensity. Actually, the much larger number of persons with osteopenia determines a significant occurrence of fractures in people diagnosed with this condition. There is no global consensus for screening patients at risk of osteoporotic fracture; however, several medical associations recommend a targeted approach to the prevention of osteoporosis based on the 10-year absolute risk of osteoporotic fracture.

The main purpose of the treatments for osteoporosis is to decrease the risk of fragility fractures. Therefore, the capacity to assess the risk of fracture is critical for the identification of patients who are eligible for intervention.^1^ The fracture risk assessment tool (FRAX), a computer based algorithm, is the most thoroughly studied and widely used tool to calculate the risk of fracture.^2^ The FRAX was released in 2007 by the World Health Organization Collaborating Centre at Sheffield, United Kingdom (UK), to estimate the individualized 10-year probability of hip and major osteoporotic fractures (hip, clinical spine, distal forearm, and proximal humerus).^3^ The FRAX tool integrates 8 clinical risk factors (CRFs): previous fragility fracture, parental hip fracture, smoking, systemic glucocorticoid use, excess alcohol intake, body mass index, rheumatoid arthritis, and other causes of secondary osteoporosis); those, in addition to age, sex and BMD at the femoral neck (an optional input) contribute to the 10-year fracture risk estimate. The probability of fracture is computed taking the risk of fracture and the risk of death into account.^3^

The FRAX is increasingly being used as a guide for clinical decision-making, and FRAX models are currently available for 63 countries and in 32 languages, covering 79% of the world population aged 50 years or older.^4^ As fracture probability differs markedly within and across regions of the world,^5^ the existing FRAX models were calibrated to the epidemiology of fracture and death in individual nations. The FRAX model for Brazil was released in 2013, and in 2015 an important paper was published, and it described the data used to develop and calibrate the Brazilian FRAX model, illustrating its features and developing intervention thresholds.^6^ The FRAX tool is the first to provide a country-specific fracture-prediction model for Brazil, and it was recently integrated into the Brazilian guidelines for the diagnosis and treatment of postmenopausal osteoporosis,^7^ adopting the approach recommended by the National Osteoporosis Guideline Group (NOGG) in the UK, in which the intervention threshold is set at the age-specific fracture probability equivalent to women with a previous fragility fracture.^8^ The NOGG management strategy requires the consideration of two additional thresholds: the lower assessment threshold (a probability below which neither treatment nor a BMD test should be considered), and the upper assessment threshold (a probability above which treatment may be recommended irrespective of BMD). Those with probabilities above the lower assessment threshold but below the upper assessment threshold should be considered for a BMD test and have their fracture probability reassessed.^8^

Since the release of the FRAX Brazil in 2013, there have been questions related to the limitation of the epidemiological studies that served as database for its calibration, as well as questions regarding whether or not those facts could diminish the relevance of the Brazilian FRAX tool in the clinical practice. Information about the epidemiology of fractures in Brazil is sparse.^9^ There are, however, several published regional reports on the incidence of hip fracture in different regions of Brazil.^10^ ^11^ ^12^ ^13^ Briefly, the calibration of the FRAX Brazil was performed using data from four Brazilian epidemiologic studies, and, as there was no consistent difference in hip fracture incidence according to region, the results were amalgamated to obtain a national data estimate on the incidence of hip fracture and mortality.^10^ ^11^ ^12^ ^13^ Those studies have been conducted in the cities of Porto Alegre (located in Southern Brazil),^13^ Marília (located in the Southeast^12^), and Sobral^10^ and Fortaleza^11^ (located in the Northeastern region of the country). While recognizing that the estimates of data on hip fracture rates representing the whole country were based on regional studies, there was no consistent difference in the results among the studies, all of which followed a robust methodology and were published in peer-reviewed international journals, and their combined results were considered representative of the epidemiology of hip fractures in Brazil by the original FRAX developers.^6^ Another frequent question refers to the fact that Brazil is a multiethnic country, and fracture rates may differ according to ethnicity. In the vast majority of available FRAX models worldwide, ethnicity could not be built into the models due to the paucity of data from which to populate any model. In fact, fracture probabilities based on ethnicity are only present in the FRAX USA and FRAX Singapore.^6^ In Brazil, the regional estimates included those from the Northeastern, Southern and Southeastern regions, which have populations of mixed ethnicity, and, again, there was no consistent difference in the incidence of hip fracture according to region, suggesting that the decision to amalgamate the regional data was reasonable.^6^

In summary, the FRAX Brazil is a free online tool that helps Brazilian clinicians to better identify women and men in need of intervention (at the highest risk of fragility fracture), and, thereby, it helps improve the allocation of our limited healthcare resources. The FRAX Brazil utilizes several known clinical risk factors rather than BMD alone to calculate a patient's 10-year fracture probability, thus making it particularly useful in parts of our country where the DXA technology is scarce or not available. Moreover, the tool is calibrated by the best available epidemiological data in our country, and can be continually upgraded as new data emerge, ensuring greater efficiency and ease-of-use in the clinical practice.
